# Artificial intelligence in chronic and autoimmune pancreatitis: diagnosis, prognosis, and personalized management

**DOI:** 10.3389/fmed.2026.1859349

**Published:** 2026-07-23

**Authors:** Xiaoming Xu, Hualei Chen, Xi Zhang, Xuetao Wang, Yuanyuan Ding, Guobin Wang, Zhaoran Zhang

**Affiliations:** 1 Department of Gastroenterology, Jining No. 1 People's Hospital, Jining, Shandong, China; 2 Department of Hepatobiliary Surgery, Jining No. 1 People's Hospital, Jining, China; 3 College of First Clinical Medicine, Shandong University of Traditional Chinese Medicine, Jinan, China

**Keywords:** artificial intelligence, autoimmune pancreatitis, chronic and autoimmune pancreatitis, diagnosis, personalized management, prognosis

## Abstract

Chronic pancreatitis (CP) and autoimmune pancreatitis (AIP) have overlapping clinical and imaging features with pancreatic ductal adenocarcinoma (PDAC), resulting in frequent misdiagnosis and improper clinical treatment, and conventional diagnostic methods are subject to subjective factors, low accuracy and sampling errors. A review of the paradigm shift brought about by artificial intelligence (AI) in the diagnosis, prognosis and individualized management of CP and AIP. Based on AI, including deep learning and radiomics, has achieved a high-precision differential diagnosis and severity grading of CP and AIP by analyzing endoscopic ultrasound, CT, MRI and other imaging modalities, and integrates multi-source clinical, serological and omics data to further improve diagnostic efficiency. AI-powered digital pathology has realized quantitative histological analysis, and prognosis AI models can help predict complications such as exocrine pancreatic insufficiency and treatment non-adherence to support early intervention. In addition, we also address the current problems of AI in clinical translation, such as model overfitting and the “black box” issue, and indicate that prospective multicentre studies, explainable AI, and multimodal data integration will be the primary directions for future research, thereby promoting the development of precision medicine in pancreatology.

## 1 Introduction

Chronic pancreatitis (CP) and autoimmune pancreatitis (AIP) are two major inflammatory pancreatic diseases. While they differ fundamentally in pathogenesis, their clinical manifestations largely overlap. CP is a progressive fibro-inflammatory disease characterized by irreversible structural damage of the pancreas. In contrast, AIP is a unique immune-mediated form of pancreatitis, most commonly associated with IgG4-related disease (1–4). Focal CP and AIP may present as a mass in the pancreatic head, causing obstructive jaundice and weight loss. Apart from mass lesions, CP causes recurrent pain, malnutrition and pancreatic dysfunction (5). AIP is divided into two types: type 1 (IgG4-related, multi-organ involvement) and type 2 (idiopathic duct-centric, pancreatic-localized, linked to inflammatory bowel disease) (4). The lesions present in a manner that is almost indistinguishable from pancreatic ductal adenocarcinoma (PDAC), and accurate differential diagnosis is required to prevent unnecessary surgery and guide appropriate treatment (6, 7). Early-stage CP is difficult to diagnose due to the lack of specific biomarkers, and a comprehensive assessment combining clinical symptoms, imaging findings and functional tests is required (8). AIP is diagnosed in accordance with the HISORt criteria or ICDC by evaluating histopathological features, imaging results, serological data, extra-organ involvement and steroid treatment response (9, 10). The diagnosis of both diseases remains difficult because no single test is sufficient in all clinical settings. Imaging examinations have subjective and qualitative limitations, are prone to overlapping imaging features, and are highly dependent on the experience of radiologists and lack of quantitative standards (11, 12). Although elevated serum IgG4 is an important feature of AIP, its sensitivity and specificity are imperfect. Relying solely on serum IgG4 may lead to misdiagnosis (13). Histological examination is the gold standard for diagnosis. But it often limited by sampling errors, insufficient fine needle biopsy materials and difficulty in preserving the complete tissue structure (14). Importantly, focal AIP and mass-forming inflammatory lesions may still be difficult to distinguish from pancreatic malignancy, which increases the risk of delayed or inappropriate treatment. Benign lesions mistaken for malignant tumors may cause extensive pancreatic resection, while misdiagnosing PDAC as CP or AIP can delay curative treatment or lead to inappropriate steroid use (6).

Both CP and AIP also require longitudinal surveillance. CP requires focused observation of organ morphology, pain, pancreatic duct complications, nutrition, and endocrine and exocrine functions of the pancreas. AIP requires monitoring disease activity, efficacy, recurrence, biliary tract and extrapancreatic complications. Long-term follow-up of CP and AIP requires multi-dimensional and systematic management. Thus, developing computational tools to help clinicians integrate complex data is of great clinical significance (8, 15).

In the context of ongoing progress in the diagnosis and treatment of inflammatory pancreatic diseases, artificial intelligence (AI) methods that combine radiomics, deep learning, and structured clinical data are increasingly being recognized as effective tools for optimizing clinical decision-making. By enabling standardized diagnosis, objective disease quantification, precise prediction of disease course, and support for precision medicine, these approaches show substantial potential for improving the clinical management of pancreatic inflammatory diseases (16, 17). This review summarizes recent advances in the application of AI to the diagnosis, disease monitoring, and prognostic assessment of CP and AIP (Supplementary Figure 1). It highlights the potential of AI to improve diagnostic standardization, increase differential diagnostic accuracy, and support objective prognostic prediction, while also discussing current challenges and future directions for clinical translation.

## 2 Artificial intelligence in CP

### 2.1 Automated diagnosis based on endoscopic ultrasound

EUS provides high-resolution visualization of pancreatic parenchymal and ductal microstructures, which is the core diagnostic modality for CP and AIP. The Rosemont standard defined US diagnostic criteria for CP (18). But this standard depended on operator experience and subjective judgment. It leaded to high inter-observer variability and poor clinical consistency, which severely restricts the popularization and stability of EUS diagnosis in primary hospitals. With the development of AI and processing technology of digital image, automated diagnostic methods based on EUS images have gradually emerged (19). The meta-analysis showed that the overall accuracy of AI-assisted EUS in diagnosing pancreatic mass (including PC, CP, AIP, etc.) was as high as 93.6% (20). The computer-aided diagnostic technology of EUS elastography based on artificial neural network (ANN) by Sǎftoiu et al. could quickly and accurately identify the benign and malignant pancreatic masses, which was better than image analysis and manual film reading alone (21). A study by Marya et al. showed that a convolutional neural network (CNN)-based model had a well sensitivity and specificity (both 81%) in differentiating CP from other pancreatic masses in EUS images (AUC 0.847) (22). The AUC of the model developed by Kuwahara et al. based on EfficientNetV2-L CNN reached 1.00 (0.61–1.00) in the validation set, indicating that it has good ability to discriminate CP (23). Compared with conventional EUS, AI-assisted EUS provides superior performance in the detection and differential diagnosis of pancreatic lesions. This advantage is mainly attributed to the ability of AI models to precisely segment the pancreas and reliably recognize Rosemont features, which reduces interobserver variability and enhances the objectivity, stability, and reproducibility of diagnosis. Contrast-enhanced ultrasound (CEUS) offered real-time evaluation of pancreatic microvascular perfusion. It provided unique value for early diagnosis, disease differentiation, and complication assessment (24). The CEUS image-based deep learning radiomics (DLR) model performed well in CP-assisted diagnosis (AUC 0.953–.986), which was better than radiologists with 3–15 years of experience, and significantly improved diagnostic sensitivity and specificity (25).

### 2.2 Diagnosis and severity grading based on CT radiomics

Deep learning and radiomics were profoundly transforming the CT-based CP diagnosis and treatment model, which improved diagnostic efficiency through automatic segmentation, objective grading, and accurate identification. Earlier studies have established a model using CT imaging features of the pancreas combined with texture feature analysis to differentiate the diagnosis of PC and CP, and showed good diagnostic efficacy (AUC = 0.98) (26). Mashayekhi et al. modeled the radiomics features of pancreatic-enhanced CT based on IsoSVM, which could better identify CP in patients with abdominal pain (AUC 0.9) (27). A large-scale multicenter retrospective study developed a semantic segmentation model based on 3D nnU-Net deep learning, which could accurately segment complex pancreatic structures in CP patients, with high segmentation accuracy and robustness, adaptability to multi-site and multi-scanner, and is better than manual segmentation in terms of labeling heterogeneity, efficiency and stability (28). AI radiomics models could automatically grade CP severity using plain CT. The CATS model performed exceptionally well in distinguishing different severity levels, with AUCs of 0.96 and 0.88, and even outperformed radiologists by identifying subtle calcifications that were missed on routine review (29). Another study used deep learning to predict pathological CP grade (CPpG) from CT images. It's deep learning radiomics score (DLRS) performed better with an AUC of 0.84 (30). CT radiomics has shown strong performance in the diagnosis and grading of CP. However, in complex differential diagnosis, AI-based multimodal fusion offers distinct advantages. A deep learning radiomics model integrating contrast-enhanced ultrasound achieved AUCs of 0.953–0.978, matching the performance of experienced radiologists (25).

### 2.3 Severity grading based on MRI omics

AI plays key roles in MRI diagnosis of CP: automatic segmentation, lesion detection and qualitative, and quantitative feature extraction. The PanSegNet model achieved high-precision pancreatic segmentation in MRI images. The Dice similarity coefficient of 80%−88%, providing a reliable tool for accurate measurement of pancreatic volume and assessment of atrophy (31). AI can automatically identify and qualitatively analyze abnormal changes such as pancreatic parenchymal fibrosis and atrophy in MRI. Based on multiparametric MRI and machine learning (ML), parameters such as ADC AND IVIM-DKI can effectively identify pancreatic masses. The perfusion fraction (f) can well distinguish PDAC and MFCP (AUC 0.84) (32). Radiomics can extract quantitative texture features from conventional MRI and quantify the changes in the microstructure of the pancreatic parenchyma, and then provid an objective basis for early diagnosis and grading of CP (33). For example, a ML model based on multiparametric MRI radiomics helped distinguish PDAC from MFCP. It reached an overall AUC of about 0.9, with good sensitivity and specificity. The diagnostic performance was clearly better than current clinical models and radiologist readings (34).

### 2.4 Integration of clinical and serological data

AI reshapes the development and application of biomarkers in CP diagnosis by integrating multi-source clinical test data. Firstly, AI has a powerful feature screening and modeling capability in single-omics data. ML models based on protein combinations such as IDH1 CAPS achieved an AUC > 0.95 for differentiating children's CP from controls in urine proteomics (35). In serum proteomics, the AUC of the XGBoost model based on markers such as NPY C1QA for differentiating pancreatic cancer from CP reached 0.84–0.92 (36). Similarly, the plasma miRNA panel-based SVM model had an AUC of 0.906 and is suitable for a wider range of people (37). In addition, AI processes complex data well, such as epigenetics and metabolomics. The plasma cfDNA methylation signature (PRKCB gene locus) constructed by the random forest (RF) algorithm had an AUC of 1.00 to distinguish CP from pancreatic cancer (38). Another study optimized the plasma metabolite pane through ML and achieved a high performance (AUC = 0.922) in a large multicenter validation (39). The integration advantages of AI were also reflected in the integration of multimodal data to improve efficiency. The fusion of RNA sequencing variants and CA19-9 deep learning models could identify resectable pancreatic cancer and CP (40). The secretory gene panel screened by transcriptomic meta-analysis achieved stable differentiation in tissue and blood samples, and the AUC of distinguishing CP from pancreatic cancer reached 0.95 (41). These studies offer a new approach for precise, noninvasive diagnosis of CP

## 3 Application of artificial intelligence in AIP

### 3.1 Diagnostic model based on CT

Radiomics extracts features like texture and shape from images could turn them into usable data, which helps diagnose AIP. Traditional CT diagnosis relies on radiologists' visual assessment of morphological changes, which cannot capture subtle texture differences between AIP and PDAC. This may result in low differential diagnosis efficiency. ML models based on CT radiomics are gradually developing from single-feature modeling to multi-feature fusion, and from model efficacy verification to clinical in-depth comparison in the differential diagnosis of AIP and PDAC. Park et al. developed their venous-phase model through the RF algorithm, which performed well with 95.2% accuracy, and much better than traditional CT's. Besides, among patients without a clear clinical suspicion of AIP, radiologist considered AIP in only 67% of cases, whereas the model achieved 89.7% sensitivity and higher specificity (42). The study of E et al. subsequently confirmed that the diagnosis efficiency was the best (AUC 0.977, accuracy of 94.8%) by combining multi-stage radiomics features of CT plain scan, arterial and venous phases. This is significantly better than the single-stage model and more accurate than the subjective diagnosis of a senior radiologist (43). Li et al. first used propensity score matching to control the bias. The radiomics scoring model they built based on this has a high degree of discrimination (AUC 0.97), which shows that rigorous research methods can indeed enhance the value of the model (44). Lu et al. advanced clinical translation by developing a nomogram that integrates radiomic and CT visual features. This model outperformed single-feature models with AUC of 0.83 and offered a visualized tool to assist clinical decision-making (11). DL, particularly CNNs, enables automatic learning of hierarchical and complex feature representations from raw images without manual feature engineering. Deep features extracted from intermediate CNN demonstrate higher predictive power. Ziegelmayer et al. reported that ML models based on deep features achieved higher sensitivity, specificity and AUC (0.89, 0.83, 0.90) than using traditional radiomic features (0.72, 0.78, 0.80). Visualization heatmaps further revealed distinct activation patterns between AIP and PDAC, and helps improve diagnostic accuracy (45). PET/CT AI models enable accurate, noninvasive identification of AIP by detecting metabolic and morphological lesion differences. The early fusion of multimodal features in the PET/CT radiomics model reached 0.93, which was better than the clinical model and subjective judgment (46). Later, Liu et al. added dynamic information at both time points to increase the AUC to 0.9668, which was significantly better than traditional clinical diagnosis (47). Wei et al. combined radiomics and deep learning features to construct a PET/CT multimodal model with an AUC of 96.4%. The model can correct misdiagnoses and demonstrate the clinical value of multi-source information fusion (48).

### 3.2 Based on MRI and diffusion-weighted imaging (DWI)

MRI has an advantage over CT when it comes to comparing soft tissues. DWI reflects tissue diffusion and help distinguish inflammatory from neoplastic lesions. AIP lesions have a high cell density and limited dispersion, resulting in lower ADC values than PDAC. ADC diagrams are more subjective when viewed with the naked eye, but AI-based radiomics combine with MRI could extract richer, more specific quantitative features and improve diagnostic accuracy. Some studies have extracted texture features from ADC maps and used SVM models to distinguish focal AIP from pancreatic cancer with 96.2% accuracy (49). This study demonstrated that a DWI-ADC radiomics model combined with an SVM AI classifier can effectively distinguish AIP from malignant pancreatic tumors and supported noninvasive diagnosis of autoimmune pancreatitis. The meta-analysis showed that the MRI AI model had better sensitivity than the CT AI model (84 vs. 59%). Their specificities were similar (97 vs. 99%). It confirmed that MRI had greater clinical advantage in detecting and differentiating this type of lesion. This result possibly due to the MRI's multiparameter imaging capabilities (50). MRI radiomics and AI showed promise for differentiating autoimmune pancreatitis, but current studies had methodological limitations, incomplete validation, and weak clinical translation evidence. Large, multicenter prospective studies were needed (51).

### 3.3 AI model based on EUS for diagnosing AIP

The AI model of EUS provides a new tool for non-invasive AIP diagnosis. The development trend is shifting from traditional ML to deep learning and from single identification to multi-classification tasks. Early models constructed by Wang et al. based on SVM and novel texture features have been able to effectively distinguish AIP from CP, with an accuracy rate of 89.3% (52). Marya et al. used ResNet50V2 to identify AIP and PDAC, achieving an AUC of 0.96. EUS interpretation was highly operator dependent and subjective, while AI-CAD enables more objective diagnosis (22). In distinguishing benign from malignant lesions, the deep learning model based on EfficientNetV2-L developed by Kuwahara et al. showed 100% sensitivity in internal validation. However, external validation revealed misdiagnoses of benign lesions, such as AIP, and decreased sensitivity to 73%. This indicates the need for further multicenter, prospective validation (23). Nakamura et al. optimized ResNet152 by combining image enhancement and temporal data. The model achieved an area under the curve (AUC) of 0.85 and an accuracy of 0.80, which is outperformed endoscopists. The overall diagnostic accuracy of endoscopists was only 63% (53). However, the retrospective single-center nature of the study means it has not yet fully met the standards for clinical deployment. The intelligent endoscopic ultrasound (iEUS) system developed by Ni et al. was based on the Mask RCNN ResNeXt-101-D architecture and could analyze images and real-time videos of various pancreatic lesions. In prospective diagnoses, the model achieved 97.0% accuracy. Its diagnostic performance is comparable to that of mid- and senior-level physicians, and it can improve diagnostic capability across all levels while narrowing performance gaps (54). Future work should include multicenter external validation, expansion of case numbers and lesion types to advance clinical translation. In addition, integrating the AI model with real-time endoscopic workflows and combining diverse clinical data will be key directions for future development.

### 3.4 AI diagnostic model based on clinical and serological data

The diagnosis of AIP is rarely based on imaging alone. AI models integrating multimodal data can demonstrate superior performance (55). Serum IgG4 is an important marker for diagnosing AIP. A multicenter study used machine learning algorithms such as random forests to analyze patient characteristics and blood indicators, and the AUC of the RF model for diagnosing IgG4-related diseases reached 0.974 after incorporating IgG4 levels (56). Although serum IgG4 is limited in diagnosis alone (some AIP patients have normal indicators), it is valuable when combined with other indicators in AI integrated models. A multicenter retrospective study using a RF model included only 6 clinical and ultrasound features (CA19-9, abdominal pain, jaundice, AIP typing, blood flow signal, morphology), External validation showed that it was comparable to or better than that of senior radiologists in diagnosing AIP (57). Additionally, a multiparameter model combining serological indicators and metabolic parameters has been constructed, including serum IgG4 levels greater than or equal to 280 mg/dl, CA19-9 levels less than or equal to 85 U/ml, and metabolic tumor volume (MTV). This model exhibits extremely high diagnostic efficacy (AUC 0.991, sensitivity 95.3%, and specificity 96.4%) (58). Beyond clinical and serologic data alone, future AI models that integrate multimodal inputs may enable earlier, more accurate, and noninvasive diagnosis of AIP (22, 48).

## 4 Digital pathology and quantitative histology

A deep-learning model such as DeepLabV3+ can be used to perform automated segmentations and precise quantifications of the tissue areas within whole-slide images of chronic pancreatitis. Using latent category analysis, a new histopathology-derived pathological grading system (CPpG) has been established that can effectively grade the severity of CP and validate the capability of AI to generate objective grading standards through quantitative histological analysis (30). QFibrosis-based AI digital pathology platforms can achieve label-free visualization of collagen fibers via SHG microscopy (59). Combining SHG microscopy with machine learning enables the acquisition of hundreds of quantifiable morphological characteristics of collagen, such as fiber length and width, network structure, and cross-linking degree (60, 61). Notably, qFibrosis accurately detects subtle fibrosis regression after therapy in liver fibrosis models (59). Applying this technique to pancreatic biopsy enables continuous and fine assessment of fibrotic burden. As a result, it can more accurately reveal the natural course of the disease and assess treatment response (62).

Significant infiltration of IgG4-positive plasma cells is the most characteristic histopathological marker of AIP. Manual reading and counting of IgG4-positive plasma cells is time-consuming and subjective, and small specimens are susceptible to sampling errors (63, 64). Deep learning can accurately and automatically identify counts, improve objectivity and efficiency, and integrate multiple features to develop diagnostic models (65). However, there are still few studies on AI combined with digital pathology for AIP diagnosis. High-quality exploration is urgently needed.

## 5 Prognostic models: predicting complications and outcomes

Conventional prognostic instruments such as the chronic pancreatitis prognosis score (COPPS) can stratify short- to mid-term hospitalization risk using pain, HbA1c, CRP, BMI and platelet count, but their predictive strength remains moderate, they are insensitive to early disease, and they correlate poorly with morphologic imaging stages (66). To address these limitations, AI has been increasingly applied in the assessment of CP. AI models dynamically predict CP complications, severity and prognosis to support early intervention. Chen et al. built a CATS model (non-enhanced CT + 3D CNN) for automatic CP severity grading, which correlated significantly with exocrine function, hospital stay, pain and readmission (29). They further established CPpG via AI-quantified pathological sections and a DLRS model (CT radiomics + deep learning); patients had 2.32 × higher diabetes risk, 1.41 × higher exocrine insufficiency risk, and significantly longer hospital stay, higher readmission rate and pain score than grade I patients (30). Xie et al. used NLP to define advanced CP from radiology reports; its 5-year mortality was 33.2%, with low body weight, smoking and diabetes as controllable independent risk factors (67).

Exocrine pancreatic insufficiency (EPI) common complication of CP. Nalliah et al. constructed an EPI prediction model using CT radiomics and XGBoost, which externally verified that the AUC was 0.80–0.81, which could quantitatively assess the degree of damage (68). Tanaka et al. used a decision tree to screen out BMI and total protein and constructed an EPI simple scoring system. The AUROC of model reached 0.782. These results demonstrate that the model is stable and serves as an effective, noninvasive, and low-cost screening tool (69). Pyenson et al. used random forests to analyze administrative claims data and identify cases of undiagnosed EPI. The model can predict EPI based on non-specific symptoms effectivly (AUROC 0.94), and the estimated prevalence is 12 times higher than the actual diagnosed prevalence, significantly improving the sensitivity and coverage of identifying complications (70).

There is still a challenge in predicting pancreatic diabetes in patients with CP. Nalliah et al. showed that the efficiency of the radiomics model based solely on CT features was limited, with an AUC of only 0.63 and an accuracy of 0.39 for diabetes prediction (68). However, the CPpG grading based on CT prediction by Chen et al. showed that the risk of diabetes in grade II patients was increased by 2.32 times, suggesting that the pathology-imaging combination strategy may improve the predictive value (30).

Manava and others. Used ML to predict non-adherence of pancreatic enzyme replacement therapy in CP patients. The AUC value of the XGBoost model reached 0.91, and SHAP analysis indicated that disease course, age, low-fat diet, rural residence and undergraduate education level were key factors. Based on this, a non-adherence (NC) score can be established for target intervention of the high-risk group (71). Although the established models are still imperfect, the systematic Development approach offers a quantifiable basis for the personalization Management of CP.

## 6 Challenges, limitations and future directions

There are still many problems that need to be addressed in AI's application to clinical settings. Most of the existing studies are retrospective, single-center designs often with relatively limited sample sizes, which may introduce selection and information bias. There is a risk of overfitting the model and reducing its generalization ability (6). Moreover, many models have only been evaluated by internal validation, and the lack of independent external validation or prospective testing means that the reported AUC, sensitivity and specificity may be optimistic and may not fully represent real-world performance across different institutions, scanners, patient populations and disease stages. Besides, the diagnostic logic of deep-learning-based models is opaque, and this lack of transparency directly reduces clinicians' confidence in the technology (72). To explainable AI technology, such as SHAP values and saliency graphs can be used to reveal what part of the algorithm has driven which results. Make the model have more credibility (73). CT scanning protocols, MRI field strengths and processor settings for endoscopic ultrasound (EUS) are all factors that can affect radiomic features, thus impacting the performance of the model (74). Truly integrating AI into the clinic also requires breaking down the barriers to interoperability between it and electronic health records and image archiving communication systems (75). Establishing a reasonable reimbursement mechanism for AI-assisted inspections is a prerequisite for ensuring fair access (72). Taken together, retrospective studies alone are not sufficient to put these tools into practical application. In the future, we need to work together and shift toward prospective, multicentre trials with prespecified protocols, harmonized clinical, serological and imaging data collection, independent external validation cohorts and subgroup analyses to test model robustness.

## 7 Discussion and conclusion

AI has brought about new opportunities for chronic pancreatitis and Autoimmune pancreatitis's therapy (Tables 1, 2). Compared with traditional methods that depend on subjective image interpretation, limited biomarkers, manual histological assessment, and empirical scoring systems, AI offers more objective, quantitative, reproducible, and multimodal evaluation. By combining the strengths of radiomics, deep learning and clinical experience. To solve the problem of early diagnosis in clinics, distinguish diseases from cancer types, and provide an individualized prognostic assessment. For instance, AI assists in interpreting endoscopic ultrasound images, grading fibrosis, and forecasting metabolic complications. It shifts the direction of development from a person's individual feeling state to an objective standard that can be reproduced in the field; it is a change in people's ways of observing things. This way, it may help lower the error rate of diagnosis and thus improve treatment plans. By integrating radiomics, deep learning, and clinical data, AI may improve early diagnosis, differential diagnosis from pancreatic cancer, severity stratification, and prognostic prediction.

**Table 1:** Diagnosis of chronic pancreatitis based on AI technology.

References	Study type	Method	AI technology	Input data	Model performance	Validation	Comparison
Kuwahara et al. (23)	Retrospective study	EUS	CNN	EUS image data	AUC 1.00	Internal validation	/
Săftoiu et al. (21)	Prospective study	EUS	CAD, ANN	Quantitative data from EUS dynamic imaging	Correct classification rate 82.95%	Internal validation	Clinicians
Marya et al. (22)	Retrospective study	EUS	CNN	EUS static images and video frames	AUROC: 0.847	Internal validation	Clinicians
Tong et al. (25)	Retrospective study	CEUS	DL	CEUS images	AUC 0.986	Internal validation external validation	Clinicians
Chen et al. (29)	Retrospective study	CT	DL	Pathological Images CT images	AUC 0.84, Sensitivity 74.03%, Specificity 85.71%	Internal validation external validation	Modeling methods
Chen et al. (30)	Retrospective study	CT	3D U-Net	Radiomics features CT imaging features clinical baseline characteristics	AUC 0.98, Specificity 96.25%, Sensitivity 91.97%	Internal validation external validation	Clinicians traditional scoring CATS, M-ANNHEIM, Cambridge
Ren et al. (26)	Retrospective study	Contrast-enhanced CT	DL	CT imaging features texture features	AUC 0.98, Sensitivity 94%, Specificity 92%, Accuracy 94%	Internal validation	/
Mashayekhi (27)	Retrospective study	Contrast-enhanced CT	IsoSVM	Imageomics features	Sensitivity 71%, Specificity 95%, AUC 0.90	Internal validation	/
Nalliah et al. (28)	Retrospective study	Contrast-enhanced CT	DL	CT imaging features	Mean dice 0.80 ± 0.15	Internal validation external validation	Clinicians
Keles et al. (31)	Retrospective study	MRI	PanSegNet	T2w MRI images	Mean DSC 0.85 ± 0.16	Internal validation external validation	Clinicians, Modeling Methods
Malagi et al. (32)	Prospective study	MRI	ANN	Quantitative parameters texture features	AUC 0.84	Internal validation external Validation	Traditional clinical methods
Deng et al. (34)	Retrospective study	MRI	SVM	Characteristics of radiomics clinical characteristics	AUC 0.893–0.997	Internal validation external validation	Clinical models
Moore et al. (35)	Cross-sectional study	Urinary Proteomics	ML	Expression levels of urinary quantitative proteins	ROC-AUC > 0.95	Internal validation	Traditional blood markers
Kim (36)	Cohort study	Serum Proteomics	LASSO elastic net + XGBoost	Serum proteomics NPY, C1QA, CDHR2, CA19-9, IFNGR2, CGB3	AUC 0.87	Internal Validation external validation	Traditional blood markers
Wu et al. (38)	Basic Research	Tissue sample testing liquid biopsy testing	SVM	DNA methylation data mrna data multi-omics data	AUC 1.0	Internal validation external Validation	Traditional clinical methods, miRNA models
Oehrle et al. (39)	Retrospective study	Serological testing	ML	Plasma metabolites serum CA19-9	AUC 0.922, sensitivity 79.6%, specificity 92.2%	Internal Validation External Validation	CA19-9 traditional scoring MxPancreasScore
Al-Fatlawi et al. (40)	Retrospective study	Peripheral venous blood	DFFNN	High-quality RNA variants clinical marker CA19-9 gender	AUC 0.96	Internal Validation	CA19-9
Cao et al. (37)	Retrospective study	Plasma sample testing	SVM	miRNA expression levels	AUC 0.906, accuracy 75.7%, sensitivity 77.1%, specificity 74.3%	Internal Validation	CA19-9
Khatri (41)	Retrospective study	Transcriptomics testing (tissue/blood)	ML	Differentially expressed secretory genes	AUC 0.95, sensitivity 78%, specificity 89%	Internal validation external validation	Traditional imaging methods CA19-9

AUC, area under the curve, AUROC, area under the receiver operating characteristic curve, EUS, endoscopic ultrasonography, CEUS, contrast-enhanced ultrasonography, CT, computed tomography, MRI, magnetic resonance imaging, CNN, convolutional neural network, CAD, computer-aided diagnosis, ANN, artificial neural network, SVM, support vector machine, DSC, dice similarity coefficient, NIfTI, neuroimaging informatics technology initiative, LASSO, least absolute shrinkage and selection operator, XGBoost, extreme gradient boosting, mRNA, messenger ribonucleic acid, miRNA, micro ribonucleic acid.

**Table 2:** AI combined with imaging data to diagnose autoimmune pancreatitis.

References	Study type	Method	AI technology	Input data	Model performance	Validation	Comparison
Ni et al. (54)	Two-way cohort study	EUS	CNN	Pixel features of EUS B-mode ultrasound (BUS) images/videos	Accuracy 97.0%, sensitivity 69.2%, specificity 98.9%	Retrospective training validation prospective clinical validation	Modeling methods, clinicians, EUS-TA
Nakamura et al. (53)	Retrospective clinical study	EUS	CNN	EUS image pixel-level features	AUROC 0.85, accuracy 80%, sensitivity 87%, specificity 63%	Internal validation	Modeling methods, clinicians
Kuwahara et al. (23)	Retrospective clinical study	EUS	DCGAN, CNN	EUS static image data	Sensitivity 73%	Internal validation external validation	N/A
Marya et al. (22)	Retrospective clinical study	EUS	CNN	EUS static image/video frame fusion data	Sensitivity 90%, Specificity 93%, AUROC=0.963	Internal validation	Clinicians
Wang et al. (52)	Retrospective clinical study	EUS	SVM	EUS image texture features	Sensitivity 84.1%, specificity 92.5%, accuracy 89.3%	Internal validation	Different texture features, Modeling methods, serological tests
Lu (11)	Retrospective clinical study	CT	ML: MLR, RF, SVM, DT	CT visual features and radiomics quantitative features	AUC 0.87	Internal validation clinical case validation	Modeling methods, clinicians
Ziegelmayer et al. (45)	Retrospective clinical study	CT	CNN	CT imaging features (portal venous phase)	Sensitivity 0.89, specificity 0.83, AUC 0.90	Internal validation	Traditional radiomics, With/without clinical variables
Park et al. (42)	Retrospective clinical study	CT	RF	CT radiomics features (arterial phase, venous phase)	Sensitivity 89.7%, specificity 100%, accuracy 95.2%, AUC 0.975	Internal validation	Traditional CT morphology, clinicians
E et al. (43)	Retrospective clinical study	CT	RF	CT radiomics features (non-contrast, arterial phase, venous phase)	Sensitivity 93.3%, specificity 96.1%, accuracy 94.8%, AUC 0.977	Internal validation	Clinicians
Li et al. (44)	Retrospective clinical study	MDCT	Radiomics Feature Extraction LASSO Logistic Regression Modeling	CT imaging quantitative features	AUC 0.97, Sensitivity 95.24%, Specificity 92.73%, Accuracy 94%	Internal validation	N/A
Zhang et al. (46)	Retrospective clinical study	PET/CT	ML: RF, AdaBoost, RBF SVM, Linear SVM	Radiomics features	AUC 0.93, accuracy 0.85, sensitivity 0.86, specificity 0.84	Internal validation	Modeling methods, clinicians
Wei et al. (48)	Retrospective clinical study	PET/CT	CNN	Preprocessed image patches: single-modal (CT, PET) and multi-modal (PET/CT)	AUC 0.964, accuracy 90.1%, sensitivity 87.5%, Specificity 93.0%	Internal validation	Modeling methods, imaging modalities, radiomics feature types
Liu et al. (47)	Retrospective clinical study	PET/CT	SVM	Pure imaging quantitative features extracted from dual-phase PET/CT	Sensitivity 85.31%, Specificity 96.04%, AUC 0.9668	Internal validation	Modeling methods, clinicians
Shiraishi (49)	Retrospective clinical study	MRI	SVM	MRI DWI-ADC maps for f-AIP/PDAC	AUC 0.962	Internal validation	Modeling methods, clinicians

AI, artificial intelligence, EUS, endoscopic ultrasonography, BUS, B-mode ultrasound, CNN, convolutional neural network, DCGAN, deep convolutional generative adversarial network, SVM, support vector machine, MLR, multiple logistic regression, RF, random forest, DT, decision tree, CT, computed tomography, MDCT, multidetector computed tomography, PET, positron emission tomography, MRI, magnetic resonance imaging, DWI, diffusion-weighted imaging, ADC, apparent diffusion coefficient, AUC, area under the curve, AUROC, area under the receiver operating characteristic curve, CI, confidence interval, LASSO, least absolute shrinkage and selection operator.

From a clinical implementation perspective, the principal value of AI lies in its capacity to augment existing workflows through the integration of imaging, EUS, serological, histopathological, and structured clinical data. In routine practice, AI models may be embedded in EUS platforms for real-time analysis or integrated into CT/MRI interpretation through PACS-compatible radiomics pipelines, thereby enabling automated feature extraction and decision support with minimal disruption to standard care. Simpler models based on ultrasound and laboratory variables may be particularly applicable in primary care settings, whereas more advanced systems may provide added value in complex differential diagnosis at tertiary centers. Successful clinical adoption will also depend on adequate clinician training and interpretable model outputs, so that AI-generated results can be appropriately assessed in the clinical context.

In the future, we need to work together and shift toward prospective, multi-center trials. As explainable and multimodal AI systems continue to develop, they will leave the laboratory and be widely applied in daily life. Updated research findings will help medical staff make clinically sound decisions moving forward. Additionally, in future research, it is necessary to explore the application path driven by artificial intelligence in the integration of radiological images, endoscopic observations, pathological sections and multi-omics data for molecular signature identification, construction of patient digital twins, prediction of disease development trends and recurrence, etc., thereby achieving precise management of pancreatic diseases and IgG4-related diseases. It is not only a technological upgrade but also brings changes to other areas. It has become an increasingly evident development direction for precise medicine in the domain of pancreatic disease.

## Supplementary material

SUPPLEMENTARY FILE 1 Search Strategy and Selection Criteria.

SUPPLEMENTARY FIGURE 1 The potential benefits of AI in the management of CP and AIP: Multimodal data, including medical imaging (EUS, CT, MRI), digital pathology, and clinical/serological parameters, are integrated and processed through AI algorithms such as deep learning and radiomics. This AI-driven paradigm yields significant clinical advantages across three main domains: (1) Diagnosis: enabling high-precision differentiation of inflammatory diseases from PDAC and facilitating automated severity grading (e.g., via AI-quantified pathological sections); (2) Prognosis: dynamically predicting long-term complications, including exocrine pancreatic insufficiency and diabetes mellitus; and (3) Personalized Management: shifting from conventional, subjective evaluation methods to objective, reproducible, and quantitative standards. Ultimately, these AI applications optimize clinical decision-making and pave the way for precision medicine in pancreatology.
