# Supplementary material for: Artificial intelligence in chronic and autoimmune pancreatitis: diagnosis, prognosis, and personalized management
Source: Front Med (Lausanne). 2026 Jul 23;13:1859349. doi: 10.3389/fmed.2026.1859349 (PMC13441912; doi:10.3389/fmed.2026.1859349)
Supplement: Supplementary file 1 [file Supplementary_file_1.docx]

Supplementary Material

**Search Strategy and Selection Criteria**

To ensure a comprehensive and transparent review of the literature, we searched PubMed, Embase, and Web of Science databases for articles published up to February 2026

The search strategy mainly employed a combination of the following keywords: ("Chronic Pancreatitis" OR "CP" OR "Autoimmune Pancreatitis" OR "AIP") AND ("Artificial Intelligence" OR "AI" OR "Machine Learning" OR "Deep Learning" OR "Radiomics" OR "Diagnosis, Computer-Assisted" OR "Image Interpretation, Computer-Assisted"). We further refined our search using terms related to clinical evaluation and management modalities, including "Prognosis," "Disease Progression," "Risk Assessment," "Decision Support Systems, Clinical," "Patient Care Management," and "Precision Medicine." We prioritized original research articles and meta-analyses that focused on artificial intelligence-based diagnosis, prognosis, and personalized management of chronic and autoimmune pancreatitis with valid clinical evaluation indicators (e.g., sensitivity, specificity, AUC).

Given that this is a mini-review, the selection criteria are "comprehensive, relevant" at the core, and all literature related to "Application of artificial intelligence in the diagnosis, prognostic evaluation and individualized management of chronic pancreatitis and autoimmune pancreatitis" are included.
